# Africa’s research publishing landscape: examining journals, publishers, and the infrastructure behind them

**DOI:** 10.12688/openresafrica.16040.1

**Published:** 2025-10-15

**Authors:** Nora Ndege, Haseeb Md. Irfanullah, Jon Harle, Tom Drake

**Affiliations:** 1 International Network for Advancing Science and Policy (INASP), Nairobi, Kenya; 2 International Network for Advancing Science and Policy (INASP), Oxford, UK; 3 Center for Sustainable Development, University of Liberal Arts Bangladesh, Dhaka, Bangladesh; 4 Center for Global Development (CGD), London, UK

**Keywords:** African research publishing, indexing, open access, publishing infrastructure, research visibility, knowledge systems

## Abstract

**Background:**

Africa’s research publishing sector is growing but remains largely fragmented and under-resourced, posing major barriers to the visibility, accessibility, and global integration of African research.

**Methods:**

This paper presents a continent-wide mapping of Africa’s research publishing ecosystem, drawing on five integrated datasets covering 1,169 publishers and 1,790 journals to assess the scale, thematic content, linguistic diversity, and the degree of openness characterising the African publishing landscape.

**Results:**

The analysis reveals that the majority of journals are published by single-journal entities embedded within universities, learned societies, and research institutes. While this decentralised model allows for locally driven publishing and alignment with national research priorities, it is often constrained by limited infrastructure, inconsistent metadata practices, and lack of professional publishing support. Geographically, publishing activity is concentrated in a few countries, most notably Egypt, Nigeria, and South Africa – reflecting disparities in research investment and infrastructure across the continent. Disciplinary patterns reveal a strong emphasis on the social sciences and humanities, shaped by post-independence academic development and limited commercial interest in these fields. The predominance of English in journal publishing enhances global visibility but risks marginalising non-Anglophone scholarship. The study also explores the increasing role of commercial publishers in improving visibility and editorial standards, while raising concerns about data control, sustainability, and long-term ownership of African research outputs.

**Conclusion:**

The paper concludes by highlighting the urgent need for coordinated, African-led investments in shared infrastructure, multilingual publishing strategies, and national indexing systems. These efforts are essential to enhance research equity, reduce dependency on external platforms, and ensure African knowledge systems are robust, inclusive, and visible in a rapidly evolving global publishing environment.

## Introduction

The ability of African countries to shape their own development trajectories depends, in part, on the strength and visibility of their knowledge systems. A robust and inclusive publishing ecosystem is essential not only for amplifying African research voices but also for ensuring that locally generated knowledge informs policy, education, and innovation across the continent. Yet, African scholars continue to be marginalised within global knowledge systems that privilege outputs from high-income countries and are shaped by entrenched inequalities in research infrastructure, funding, and visibility ^1– 3^.

Despite accounting for over 17% of the world’s population, Africa contributes only approximately 2% of global research output, though this is rising rapidly, with a doubling of research output between 2015 and 2020 ^4^. Africa accounts for less than 1% of global research and development (R&D) expenditure, a stark contrast to regions such as Europe (21%), Asia (~46%), and North America (~29%) ^5^. This underrepresentation is not merely statistical but deeply rooted in systemic inequalities, financial constraints, and legacy structures that marginalise African research and diminish local scholarly agency ^6^.

The dominance of Global North institutions in shaping knowledge production has deep historical roots. Colonial models of education and research imposed Eurocentric standards that continue to influence which ideas are legitimised and disseminated ^7, 8^. These patterns were later reinforced through foreign aid regimes and development partnerships that privileged Western ways of thinking ^9, 10^. As Ndlovu-Gatsheni ^11^ points out, these deep-rooted imbalances still shape whose ideas are heard, valued, and shared globally.

One major consequence of these dynamics is the invisibility of African journals within global discovery and indexing systems. Journals published in sub-Saharan Africa are significantly less likely to be indexed in Scopus or Web of Science (WoS), compared to European journals, and are disproportionately excluded if not published in English ^12^. This limits their discoverability, citation, and perceived legitimacy.

Meanwhile, the costs of publishing in high-impact, open access venues remain prohibitive for many African researchers. The average Article Processing Charge (APC) exceeds US$3,000, with some prestigious journals charging as much as US$12,290 ^13^ – equivalent to nearly two months’ salary for an academic researcher in South Africa – and in countries like Kenya, such APCs may represent between one to six months of a researcher’s income depending on their seniority and institution ^14^. These high fees often force scholars to forfeit content ownership and agency, further entrenching asymmetries in knowledge production ^15, 16^.

Recent years have seen renewed momentum toward developing African-led, open access infrastructures. ^1^ Initiatives such as African Journals Online (AJOL), African Open Science Platform (AOSP), Open Research Africa (ORA) ^3, 14^ and institutional repositories are creating pathways for localised dissemination and greater visibility of African research ^18^. These efforts mirror regional strategies in Latin America and Asia, where platforms like SciELO and Redalyc have advanced public investment, editorial capacity, and multilingual access to promote visibility ^19^.

Nevertheless, Africa’s research publishing infrastructure remains fragmented and underdeveloped – a long-recognised challenge that continues to limit regional research visibility and coordination. Over a decade ago, Tijssen ^20^, highlighted the urgent need for an integrated journal-based information system such as a regional citation index to fill critical knowledge gaps and enable more accurate assessments of national and institutional research performance. While this call is relevant, progress towards establishing such infrastructure has been slow and efforts to establish national open access strategies or a continent-wide indexing system have yet to gain sufficient traction. The lack of metadata interoperability has hindered the development of a resilient publishing ecosystem ^21^. Publishing continues to be dominated by universities and academic institutions operating with minimal institutional support or global reach ^14^, in sharp contrast to large, Northern-based commercial publishers that dominate major indexing databases ^22, 23^.

While bibliometric platforms like Scopus, WoS, and Dimensions provide useful global comparisons, they offer only a partial view of Africa’s research publishing landscape ^24^. These databases systematically under-index African journals, overlook non-English publications, and fail to capture many university-based or society-led publishing initiatives. They also obscure the richness of interdisciplinary work and knowledge published in local languages, reinforcing narrow definitions of research excellence that penalise Global South scholarship. This concern has prompted calls for alternative metrics and the establishment of regional or national citation indexes that better reflect local relevance and epistemic diversity ^19^.

Currently, no comprehensive index or regional repository captures the breadth of African research publishing. Previous analyses have mapped parts of the landscape such as AJOL’s listings or Directory of Open Access Journals (DOAJ)-registered journals, but these efforts remain fragmented ^25^. A recent study on regional publishing models by 19 compared Europe and Latin America, emphasising the role of infrastructure, governance, and institutional incentives. However, Africa was absent, underscoring a significant evidence gap.

Studies by Tijssen ^20^ and Mouton ^26^ have long warned that African research publishing suffers from weak editorial standards, low indexing rates, and insufficient policy support. The influence of legacy systems and international prestige incentives has also pushed researchers toward Global North journals, often at the expense of local dissemination. This study seeks to address these gaps by systematically mapping Africa’s research publishing ecosystem using a multi-source methodology.

## Methodology

We conducted a systematic, multi-source mapping of African research publishing to examine the landscape of publishers and journals across the continent. The study addresses four interrelated research questions (RQs):

- RQ1: What is the current size and geographic distribution of the research publishing sector in African countries?
- RQ2: What kinds of research disciplines do African research platforms publish?
- RQ3: What languages do African research publishers publish in?
- RQ4: How open or closed is African research publishing?

These questions collectively frame the study’s focus on scale, content, linguistic diversity, and openness within Africa’s publishing landscape.

### Data sources and publisher identification

We first compiled a list of publishing entities operating within the continent, defining research publishers as organisations, institutions, or consortia that support the production and dissemination of peer-reviewed journals, whether in print or digital form. These included university presses, professional societies, national repositories, and journal platforms. We drew on five key data sources: (i) DOAJ; (ii) AJOL; (iii) African Journals, Universities and Research (AFJUR); (iv) Clarivate Analytics WoS; and (v) a proprietary dataset compiled by the University of Witwatersrand. Each source captured different segments of the African publishing ecosystem (see Supplementary Material 1). For example, DOAJ’s scope and emphasis is on open access journals; AJOL acts as a regional aggregator for African journals that opt into its platform; and Clarivate Analytics indexes journals included in its WoS database, selecting them based on citation impact and editorial standards.

To be included in our dataset, a publisher had to be physically based in Africa or demonstrate an institutional affiliation within an African country. This criterion was particularly important in the case of Clarivate Analytics’ WoS dataset, where several journals were hosted by international publishers but remained institutionally rooted in the continent. We determined institutional affiliation through the editorial office location, sponsoring institution, or publisher address. This allowed for the inclusion of journals managed in Africa but hosted by international publishers. For example, ‘Ain Shams Engineering Journal’, although published and hosted by Elsevier in the Netherlands, remains editorially anchored (e.g. peer reviewed) in Egypt through Ain Shams University. Similarly, the ‘African Journal of Urology’, now hosted by Springer, retains its institutional roots in Africa and is still listed on AJOL. In such cases, we assigned the journal to its African country of origin, prioritising editorial and institutional affiliations over hosting location. This allowed us to more accurately reflect the African footprint within global publishing databases.

As part of data structuring, we merged the datasets and extracted key attributes of each publisher: ‘publisher name’, ‘country’, ‘number of journals’, ‘articles (from the African journal dataset)’, ‘articles (from the publisher dataset)’, ‘citations (from the publisher dataset)’, and ‘count of journals’. Two researchers independently reviewed the merged data. Manual verification was conducted to confirm URL validity, active status, and consistency across indexing claims. Publishers operating under different names but managing identical journal portfolios were merged based on International Standard Serial Number (ISSN) and organisational affiliation.

The combined dataset was manually cleaned to eliminate duplicate entries, harmonise institutional naming conventions, and verify active publishing status.

### Publishing volume and characteristics

We conducted descriptive analysis of publishing volume and characteristics focusing on counts of publishers, journals, articles, and citations per entity. We used metadata from DOAJ to classify journals as: (i) fully open access where all content is freely available without embargo; (ii) hybrid access where some articles are open and some only accessible to subscribers; and (iii) subscription-based where all content is paywalled. For thematic classification, we extracted disciplinary metadata, where available, using the DOAJ classification. When thematic tags were missing, we used journal titles, aims/scope descriptions, or indexing classifications to infer broad categories such as health sciences, agriculture, education, engineering, or social sciences. We also captured language and regional data to assess multilingualism and geographic reach. Metadata from Clarivate Analytics helped identify African journals hosted by international publishers.

### Study limitations

This study has several limitations. First, our analysis was based primarily on a publisher-level dataset. While the dataset includes journals published in English, French, and Arabic, it likely underrepresents those published in indigenous or less widely spoken African languages, particularly those with limited digital presence or discoverability. Additionally, journals were only included if a corresponding publisher name could be identified. In cases where a journal was found but its publisher could not be verified or was missing from the source, that journal was excluded from the final dataset. This may have led to omissions, especially of smaller or independently hosted journals. Second, the variability and inconsistency of metadata across platforms posed a challenge; many African journals lack standardised information such as ISSNs, structured abstracts, or reliable indexing records, which restricted our ability to extract thematic and citation data with precision. Third, our reliance on secondary data from registries such as DOAJ and AJOL means that the analysis reflects the limitations of those databases, including potential lags in updating, divergent inclusion criteria, and incomplete coverage. Finally, the dynamic nature of journal lifecycles introduces temporal inconsistencies; some journals listed as active may not have published in recent years, while others may be omitted due to recent launches or irregular publication patterns. As a result, our categorisations of journal status may not fully capture real-time activity across the continent’s publishing landscape. Future research could explore incorporating ORCID-linked editorial data, author affiliations, and usage statistics to enrich the mapping of the publishing ecosystem.

## Results

### Overview of publishers in Africa

**Figure 1.**
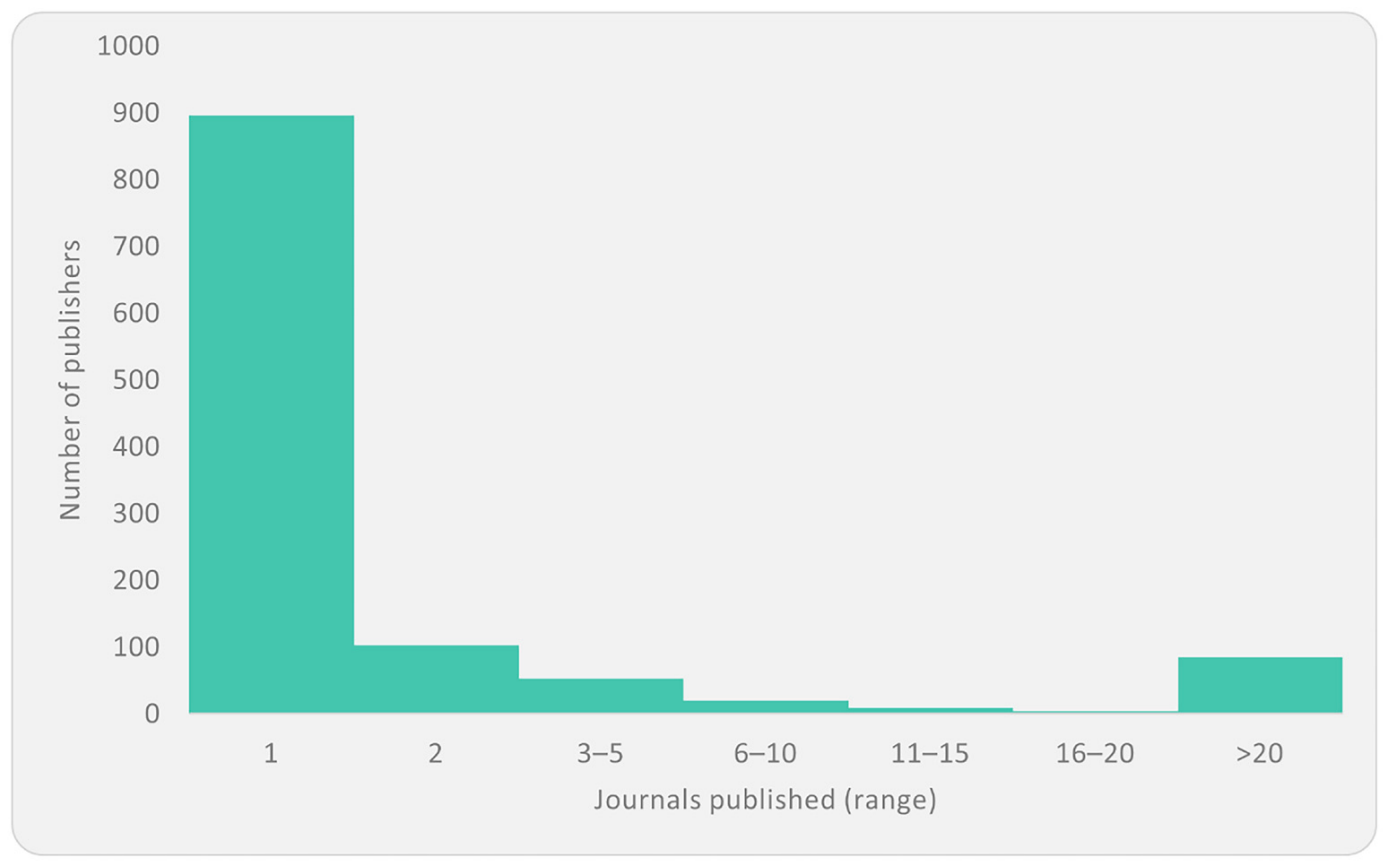
Distribution of publishers by the number of journals.

A total of 1,169 unique publishers were identified in relation to the continent, publishing 1,790 journals. The number of journals per publisher varied significantly but was heavily skewed toward publishers with very few journals. Here, 90% of the publishers publish five or fewer journals, where 77% publish one journal each and 9% publish two journals each (see Figure 1).

Gaps in publicly available data was a big challenge, with 7% of publishers not providing a figure for the number of journals that they publish. In addition, 89% of publishers lacked publicly available article counts, and out of the remaining 11%, most of them (7%) stored 1,000 or fewer articles, with only two publishers reporting a very high number of articles: 44,063 and 63,117, respectively. Similar gaps were observed on article and citation numbers (see Supplementary Materials 2 and 3).

### Geographic distribution of publishers and journals

Approximately 97% of the 1,169 publishers were based in 43 African countries or territories. The remaining 3% of publishers were without clear country information or were based outside the continent. Publishing activity is heavily concentrated in a few regions, with Northern Africa leading with 446 publishers and 506 journals, followed by Western Africa (295 publishers; 608 journals) and Southern Africa (212 publishers; 387 journals). Eastern Africa contributes 156 publishers and 228 journals, while Central Africa remains the least represented, with only 24 publishers and 23 journals. An additional 36 publishers and 38 journals were either based outside Africa or had no clear country affiliation.

**Figure 2.**
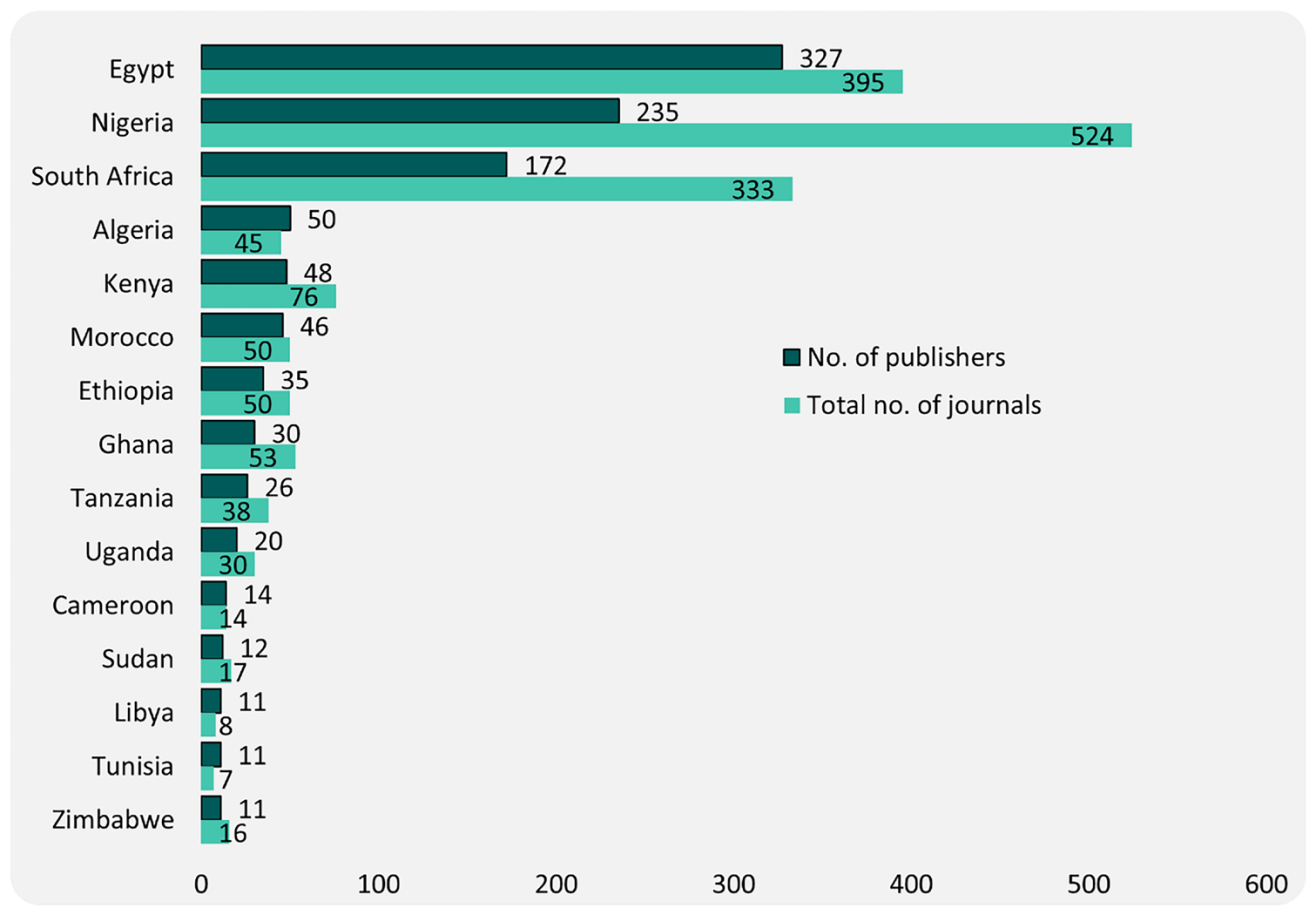
National level distribution of publishers and journals.

To better understand national-level distribution, we highlight countries with 10 or more publishers, organising them into four bands based on publisher count: 10–19; 20–49; 50–99, and 100+ ( Figure 2). This stratification allowed us to explore the relative concentration of publishing infrastructure and activity across African countries.

Three countries stand out in the highest band (100+ publishers). They include Egypt (327), Nigeria (235), and South Africa (172). While Egypt leads in the number of publishers, Nigeria publishes the most journals (524), followed by Egypt (395) and South Africa (333). The second tier (50–99) of publishers includes Algeria (50), the only country in North Africa besides Egypt to appear in this range. In the 20–49 publishers band, we find Kenya (48), Morocco (46), Ethiopia (35), Ghana (30), and Tanzania (26). Kenya and Ghana also feature prominently in journal output in this band.

Cumulatively, six countries – Egypt (28%), Nigeria (20%), South Africa (15%), Algeria (4%), Kenya (4%), and Morocco (4%) – account for over 70% of African publishers. In terms of journal numbers, Nigeria (29%) had the greatest number, followed by Egypt (22%), South Africa (19%), Kenya (4%), and Ghana (3%). The high number of publishers and journals in these countries is correlated to a high number of active university-based and society publishers.

### African journals hosted outside Africa

We identified 95 journals that are affiliated with African institutions but hosted by publishers headquartered outside the continent. As shown in Table 1, the top host countries were the United Kingdom (54), United States of America (18), India (10), and the Netherlands (10), followed by Germany, Switzerland, and Sri Lanka.

**Table 1.** List of journals with publisher’s address and publisher outside Africa.

Country	No. of external publishers
Germany	1
Sri Lanka	1
Switzerland	1
India	10
Netherlands	10
United States of America (USA)	18
United Kingdom (UK)	54
Total no. of publishers with address based outside Africa	95

Country-specific examples illustrate this: Egypt had the highest number of externally hosted journals, with 45 of their journals published abroad, primarily in the USA (17) and the UK (15); South Africa followed with 41 such journals, with the majority hosted by Taylor & Francis (UK); Nigeria had five journals hosted in India; and Kenya, Rwanda, and Tunisia each had one to two externally hosted journals (see Supplementary Material 4).

### Indexing and visibility of the African journals

An analysis of 1,790 journals revealed considerable variation in indexing coverage, with nearly half lacking any clear metadata on visibility. Among journals with available information, 665 were indexed in DOAJ and 272 appeared in Google Scholar. However, 890 journals (50%) had no indexing data available, reflecting significant gaps in metadata. Journals hosted by established platforms or through international collaborations, particularly those affiliated with Egyptian institutions but published by Elsevier, demonstrated broader indexing reach. For example, the ‘Alexandria Journal of Medicine’ was indexed in Clarivate’s Emerging Sources Citation Index, DOAJ, AJOL, and several global library directories.

### Types of publishers

Universities – including their faculties and departments – represented the largest share of publishers (478 out of 1,169). In many cases, individual schools or faculties operate as independent journal publishers. For example, in Egypt, Cairo University had 14 such publishers, Ain Shams University had 16, and Alexandria University had 19. University presses, though present, were too few to form a distinct category and were grouped under universities. Non-university academic institutions – a smaller category consisting of colleges, teaching hospitals, and defence academies – accounted for 33 publishers. Learned societies and professional associations comprised the second largest group with 350 publishers. This grouping also included related entities such as a few trusts, foundations, consortia, and federations. Research institutions and research centres, many of which operate autonomously from universities, made up the third largest category (177) of publishers and included a few research councils, laboratories, working groups, forums, and networks. There was also a small number of large-scale commercial publishers and service providers (75), together with a small collection of non-profit Diamond Open Access publishers represented by the 236 journals in DOAJ that do not charge APCs. Other publisher types included government agencies (14), museums (seven), unions (six), banks and international organisations (six), and science academies (four).

### Disciplinary focus

**Figure 3.**
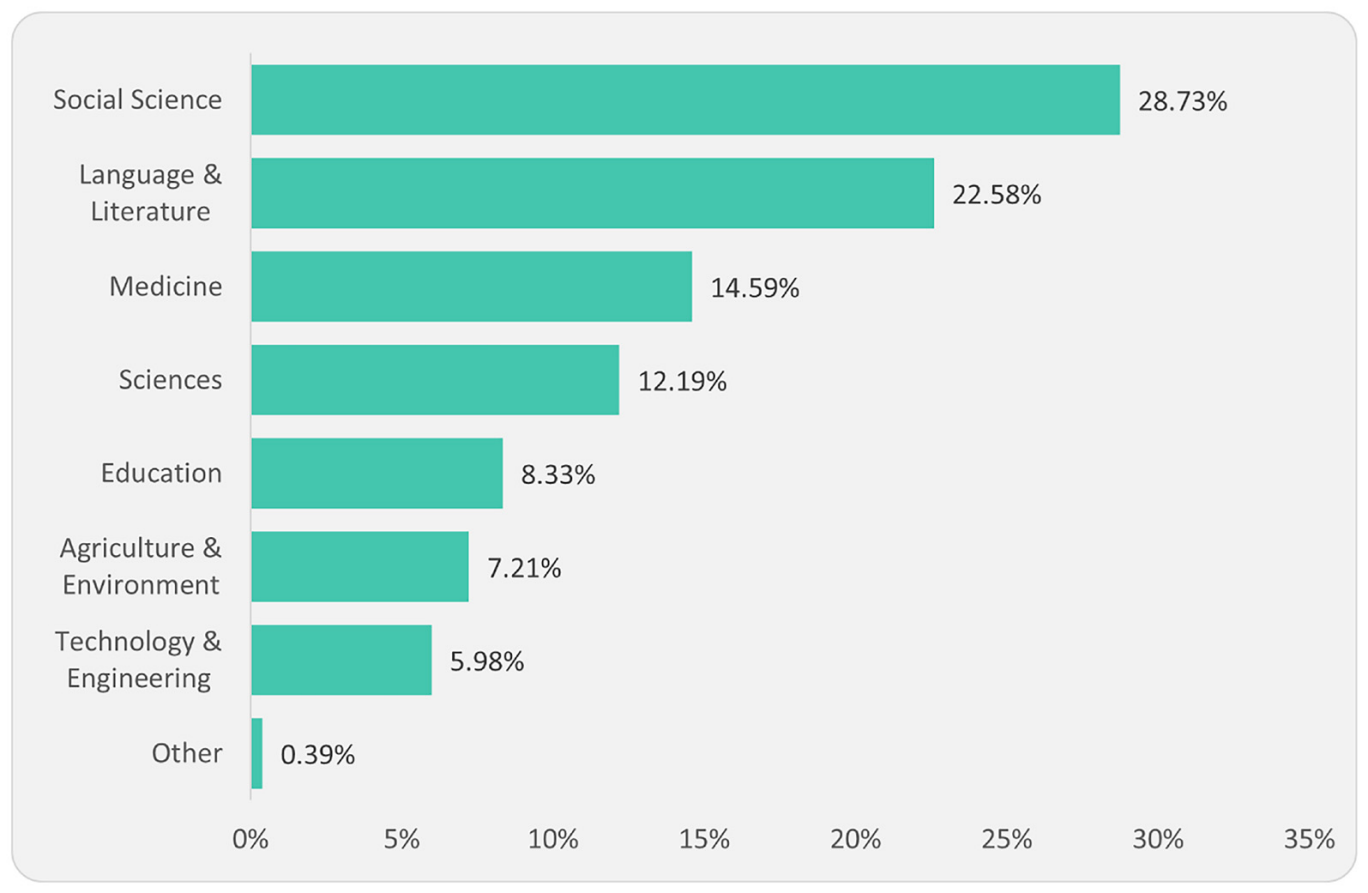
Distribution of journals by major academic disciplines (1,790).

Based on our journal dataset, an analysis of disciplinary distribution revealed that social science and related disciplines dominated the journal subject areas, followed by language and literature, medicine, and science ( Figure 3).

### Access models

Our main source of information on research access was the DOAJ. Out of 667 African journals indexed in the DOAJ, 64% (429 journals) operate under a Gold Open Access publishing model, where articles are freely accessible immediately upon publication and authors typically pay APCs. These APCs ranged from US$9 to US$1,590 with a median of US$250. These are generally modest amounts, aimed at covering basic operational costs. The remaining 238 journals (35%), follow a Diamond Open Access model, providing immediate open access without charging authors any APCs.

### Language use

On language use, 66% of DOAJ-listed journals were single-language journals. Of those, 60% were in English, 5% were in Arabic, and 0.6% were in French. Among the multilingual journals (34%), 24% of these were bilingual, mostly in English and French, or in English and Arabic. Our analysis also found that 8% of journals published articles in three languages, 2% in four languages, three journals in five languages and one in eight languages. Across all journals on DOAJ, English was the most dominant language accepted by 94% of the journals, followed by Arabic (26%), French (18%), Afrikaans (3%), German (3%), and Spanish (2%). Other publishing languages included Dutch, Zulu, Portuguese, Amharic, Chinese, Hausa, Xhosa, Italian, Persian, Swahili, Southern Sotho, Turkish, and Venda.

## Discussion

### Scale and fragmentation of African research publishing

There is a significant African research publishing sector comprising at least 1,169 separate publishers and 1,790 journals, similar to the number of publishers in Poland (1,739) or Brazil (2,013) ^19^. This sizeable output underscores a vibrant, if sometimes struggling, research communication ecosystem on the continent, characterised by a large number of small-scale university-linked publishing initiatives plus a small number of large-scale commercial publishers concentrated in a few countries.

This prevalence of academic and non-profit entities – often one-journal operations – indicates a largely decentralised, small-scale industry structure. This means that the sector is not dominated by oligopolistic publishing as seen in Europe and North America, and there are certain advantages to local institutions acting as outlets for locally produced knowledge in that they can directly curate, disseminate, and maintain control over knowledge production relevant to their own contexts. However, fragmentation of the current landscape may potentially impede quality and professional publishing capabilities, and the ability to benefit from the economies of scale ^23^. With few journals per publisher, resources such as editorial expertise, infrastructure, and funding are thinly spread. Unlike regions with consolidated national platforms or large publishers, this highly decentralised African publishing landscape raises critical questions on how to strengthen its small-scale publishers, and/or whether consolidation and shared infrastructure might be necessary to enhance quality and global reach.

The predominance of single-journal publishers is likely driven by a combination of factors, including: i) the need to create an avenue to publish society members' or faculty members’ research; ii) the academic norm that a society or faculty should have a journal; iii) the limited capacity or responsiveness of disciplinary journals to local research needs; iv) the importance of having accessible publishing avenues needed for authors’ recruitment, promotion, tenure, or scholarship; v) internal institutional pressures to provide publication channels for their academic communities (given the exclusionary tendencies of the global publishing system); and vi) rivalry between research groups or emerging research fields contributing to the creation of new journals, in some cases ^27^. Together, these factors contribute to a highly decentralised publishing landscape, where the proliferation of single-journal operations reflects local academic needs and constraints – yet is also shaped by broader global inequalities in publishing access and recognition ^28^

Despite the dominance of universities, learned societies, and research institutions in Africa’s research publishing landscape, a small proportion of African journal publishers (75 out of 1,169 publishers) are commercial publishers and service providers. This limited number of actors are playing a key role in shaping the visibility and standardisation of research outputs by offering professional editorial services; applying standardised publishing workflows; providing advanced technical infrastructure; and providing access to global distribution networks – features that many local publishers struggle to provide ^16^. Their presence is particularly relevant in countries with more mature publishing ecosystems, such as South Africa and Egypt, where journals may partner with international publishers to increase visibility and indexing. However, the relatively small footprint of commercial publishers also reflects the broader structure of African publishing, where journal production remains highly decentralised and mission-driven, rather than market-oriented. While commercial publishing has the potential to support professionalisation and scale, there are structural risks of relying on international commercial models. As demonstrated in the Nigerian analysis by Mills *et al.* ^29^, and in other cases such as Taylor & Francis in South Africa, or Elsevier in North Africa, global commercial publishers often absorb successful African journals into their portfolios – sometimes at the expense of long-term sustainability or local ownership. Without safeguards, public and institutional investments aimed at strengthening African journals could inadvertently funnel these journals into the control of global publishing giants. This underscores both the potential and the limits of commercial publishing as a vehicle for scaling African research output, highlighting the need for stronger public and institutional investments to develop regionally rooted commercial models that prioritise sustainability and local control over profit extraction.

### Regional concentration and dominant countries

Even to refer to “African research publishing” is in someways misleading, since publishing on the continent is largely concentrated in Egypt, South Africa and Nigeria, as also shown in 24. These countries stand out as continental hubs for research publishing, with each hosting more than 100 journal publishers. Their prominence likely reflects a combination of factors including stronger higher education systems, larger research budgets, their comparatively larger populations, as well as their long history in publishing. Beyond the ‘big three’, there is an ‘emerging’ middle tier of countries with noteworthy publishing activity. Algeria is one example in North Africa, with 50 publishers identified. Others that include Kenya, Morocco, Ethiopia, Ghana, and Tanzania are demonstrating sustained publishing capacity, albeit at a smaller scale (20–50 publishers). The presence of this middle group hints at a gradual widening of the African publishing landscape.

Egypt’s and South Africa’s dominance may also be partly due to their publishing integration with international indexing systems like Scopus and WoS, as well as partnerships with commercial publishers like Elsevier and Taylor & Francis. In South Africa, national research policies – including the Department of Higher Education and Training subsidy schemes – financially incentivise academics to publish in accredited high-quality journals, which boosts overall output and creates a market for strong local journals able to meet these standards ^30^. However, unlike Latin America – where countries like Brazil and Mexico have built their regional publishing platforms such as SciELO and Redalyc through public investment ^19^ – Africa’s publishing landscape has largely evolved through decentralised, small-scale efforts managed by university departments or professional societies with limited resources. Where visibility is achieved, it is often through collaborations with commercial publishers rather than coordinated public infrastructure. To further explore these dynamics, we spotlight Egypt, which has the largest number of publishers on the continent (327).

While this pattern, reflected in Box 1, signifies an attempt by African research communities to integrate with the global publishing arena for enhanced visibility, professional editorial support, and inclusion in major indexing systems, there are other concerns that these models raise. When journals shift off-continent, so does control over editorial policies, access models, and revenue flows, often at the expense of local ownership and reinvestment in African research systems. Increasingly, the issue of data control is becoming just as critical. As publishers enter into large-scale deals involving artificial intelligence and data analytics, journal metadata and user behaviour patterns are being monetised as valuable assets ^31^. This trend further compounds the risk of external dependency, as it centralises both financial and informational power in the hands of global publishing firms. Additionally, this dynamic might concentrate influence and citations in the externally hosted journals, potentially diverting attention from the bulk of home-grown journals. As a result, what may begin as a strategic move for international integration can, over time, foster deeper academic dependency where some African journals may feel the need to ‘exit’ local infrastructure in favour of established publishers outside Africa.

**Box 1. Spotlight on research publishing in Egypt.**

Among the 396 journals affiliated with Egyptian institutions, 51 are hosted by publishers outside of the country. This pattern reflects the country’s decentralised publishing ecosystem, with individual faculties and departments functioning as independent publishing entities, as opposed to being coordinated through central university presses or national infrastructure. For example, Alexandria University hosts approximately 19 such entities, while Ain Shams University accounts for 16 such publishers. These institutions often collaborate with international publishers for hosting and distribution. For example, the ‘Annals of Agricultural Sciences’ journal, although produced by the Faculty of Agriculture at Ain Shams University, is hosted and peer-reviewed on Elsevier’s platform. Similarly, the ‘Alexandria Journal of Medicine’ is hosted by Elsevier and included in multiple indexing services such as the WoS, DOAJ and AJOL, demonstrating a hybrid publishing model combining local editorial oversight with international infrastructure.

### Disciplinary focus

The dominance of social sciences, literature, language, and education journals (60%) in African countries is notable. However, comparatively low numbers of science, technology, engineering, and mathematics (STEM) journals show a significant deviation from the global data. For example, Clarivate ^32^ Journal Citation Reports 2025 indexed 14,591 science journals, 7,559 social science journals, and 3,368 arts and humanities journals (data as of July 8, 2025). In Scopus, 47,300 journals are listed in the Source Title List (May 2025 version, including discontinued titles; Elsevier, ^33^). Many journals fell under more than one high-level category, which makes the total count 56,377. 16,459 journals were categorised as social sciences (29.2%), 8,449 (15%) as life sciences, 15,877 (28.2%) as physical sciences, and 15,592 (27.7%) as health sciences.

This disciplinary deviation can be traced to the academic legacy of many African countries. Here, departments of social sciences, and arts and humanities were traditionally prioritised during the establishment of universities ^34^ which, in turn, guided research, and research publishing over the last few decades. In leading publishing countries such as Nigeria, Egypt, and South Africa, the structure and evolution of their tertiary education systems played a significant role in influencing the disciplinary composition of their publishing industries. For example, early and sustained investments in the social sciences and humanities in these countries contributed to a publishing landscape with a marked concentration of journals in these fields ^35, 36^ This trend is further rooted in colonial legacies which continue to shape institutional priorities and academic practices ^35^. As van Bellen *et al.* (2025) ^37^ also observe, in regions such as Latin America, Eastern Europe, Central Asia, and parts of North Africa, less than 30% of social science research articles are published via commercial publishers, signalling a preference for alternative, often ‘small’ locally managed publishers such as university-based or society-led journals. Africa appears to follow a similar trajectory, where journals hosted by universities and societies serve as the primary outlet for research dissemination in the social sciences. This helps explain why commercial publishers, who typically focus on STEM fields, have a relatively limited footprint in African research publishing.

### English dominance and limited multilingualism

A recent analysis of DOAJ's 17,564 journals (as of July 2023), showed that 65% were published in one language, and the remaining 35% were published in two to as many as 16 languages ^38^. Our finding is similar, since single-language African journals accounted for 66%. English, however, showed more dominance among African multilingual journals than global ones. For example, in our analysis, we found that none of the 229 bilingual/multilingual journals excluded English manuscripts, with only one exception – a journal published in Portuguese and Spanish. In comparison, the proportion of journals that do not accept English submissions is 2% globally in the DOAJ ^38^, and just 0.4% in Africa – according to our analysis.

This predominance of the English language reflects both practical and historical factors. Most of the universities on the continent were established during the colonial period and modelled on British institutions, with English institutionalised as the primary language of instruction and academic exchange ^22, 39, 40^. This legacy has persisted, reinforced by global dominance of English in research, and it continues to be favoured because it maximises international visibility, citation potential, and access to global indexing systems.

This linguistic dominance, however, poses potential trade-offs. The heavy reliance on English can limit the accessibility of research to local practitioners and communities more comfortable with Arabic, French, or indigenous African languages ^41^. Our data showed relatively few journals published solely in French or Arabic, despite large Francophone and Arabophone populations, suggesting that researchers may choose English for visibility, or that local-language journals face systemic barriers to gaining recognition. The scarcity of multilingual journals also highlights limited efforts to bridge linguistic divides, which may hinder the cross-pollination of ideas across regions. As African research publishing evolves, the structural tension between global visibility and local accessibility remains a critical issue for policymakers and publishers to address.

### Open access models and APC practices

Our results highlight the growing prominence of open access publishing across the African continent. The significant proportion of journals practicing Gold Open Access (65%), primarily funded through APCs, points to increasing alignment with global open access models; while the rest, which do not charge APCs, reflect a commitment to more inclusive publishing practices – particularly in contexts where research funding is limited. This split reveals a few important structural patterns: the inclusion of African journals in DOAJ suggests expanding adherence to open access principles; and it also indicates a growing awareness among publishers that removing paywalls can enhance the visibility and societal impact of African scholarship, especially for readers and institutions with limited access to commercial databases.

Unlike the high APCs charged by major commercial publishers, often in the range of US$1,500–US$3,000 or more per article in Europe/North America ^13^, African journals tend to levy much lower fees. These APCs are often minimal, intended primarily to cover basic publishing costs rather than to generate profit. In many cases, African journals operate with volunteer editors and have in-kind support from universities, so a median APC of around US$250 is a fraction of global rates, aimed at cost-recovery. At the same time, the substantial fraction of Diamond Open Access journals (35%) indicates that a significant portion of African publishing relies on subsidies or institutional support, instead of charging authors. The current mix of low-APC Gold and Diamond Open Access suggests an ongoing experiment in finding the right balance between openness, cost recovery, and quality.

### Infrastructure gaps and the future of African publishing

As shown in our analysis Africa’s publishing infrastructure remains unevenly developed, with the ‘big three’ countries dominating both journal production and essential infrastructure like digital repositories, journal platforms, and aggregators. South Africa and Kenya, for example, host the highest number of institutional repositories on the continent – approximately 40 and 32 respectively – providing foundational infrastructure for digital publishing and open access dissemination ^42^. South Africa also supports SciELO SA, a national indexing and publishing platform integrated into global citation networks. In contrast, many other countries lack the repositories, journal management systems, and indexing services necessary to support discoverable publishing ^18^.

Several multi-journal platforms and infrastructure initiatives have attempted to bridge these gaps. Aggregators such as AJOL and Sabinet provide regional visibility, while platforms like AfricArXiv and ORA experiment with innovative models such as preprints and post-publication peer review ^3^. However, most of these efforts still rely on external infrastructure or funding, raising concerns about long-term sustainability and control. For instance, AJOL uses the open-source software Open Journal Systems (OJS), developed and maintained by the Public Knowledge Project (PKP) in Canada, while SciELO South Africa adapts Latin American-developed infrastructure designed to support regional research publishing. In contrast, platforms like ORA rely on proprietary infrastructure developed and owned by commercial actors such as Taylor & Francis, raising concerns about long-term sustainability, ownership, and control. While newer continental initiatives such as AOSP and Africa Research Connect signal growing momentum toward African-led infrastructure solutions aiming to support researcher visibility, data-sharing, and policy harmonisation across borders, these remain early in development and require sustained investment and coordination ^18^. This limited and uneven access to high quality publishing infrastructure implies that many journals operate with minimal technical support, often relying on part-time editors and outdated systems. In countries like Nigeria, for example, the high number of journals is not matched by metadata standards or repository integration, thus restricting visibility. 

A further systemic challenge revealed in this analysis is the significant data gap across the African publishing ecosystem. Key information such as the total number of journals and articles a publisher publishes, citation data, and the location of publishers in our dataset was incomplete or missing. Such a data gap could happen for various reasons. For example, data that researchers seek, may be stored in spaces which are kept hidden by the data owners (the publishers) or their service providers. This may occur because the aggregators that the researchers use as data sources are unable to collect that data from individual publishers. Aggregators may also face challenges as a result of: journal administrators not providing consistent metadata; a limited workforce; infrastructural issues; a lack of regular updating as original funding ends; a change in ownership; and members becoming inactive. We identify this primarily as a systemic issue influenced by institutional priorities, lack of funding, and platform limitations.

These disparities highlight the need for coordinated investment in African-led platforms, national indexing systems, and sustainable metadata practices. The example of Latin America – where public and regional investment has successfully built robust, non-commercial infrastructure, such as SciELO and Redalyc – demonstrates that it is possible to develop impactful systems outside of the Global North model ^19^. Without similar investment in Africa, countries with high journal output may remain poorly visible, while those with promising infrastructure risk being underutilised.

## Conclusion

Africa’s publishing is highly decentralised, largely academic-led and fragmented in both structure and visibility. This decentralised model provides an opportunity for local ownership of research dissemination and alignment with institutional priorities. This, however, constrains professionalisation, scalability, and integration into global indexing systems. Since the publishing landscape remains predominantly mission-driven rather than market-oriented, a small number of commercial publishers are not sufficient to address structural gaps across the continent. Emerging hybrid models, where university-based publishers use international hosting platforms, offer promising pathways, but also raise concerns about sustainability, dependency, and the externalisation of control over African research content and revenues. Further, reliance on external infrastructure and inconsistent funding undermines long-term resilience of aggregators and other digital platforms.

The dominance of a few countries in the publishing space reflects stronger research investments, infrastructure, and institutional maturity. The low representation of large parts of the continent, however, mirrors broader gaps in national R&D investment, policy coordination, and institutional capacity. While the STEM fields dominate global indexed publishing, the continent’s journal publishing trends reflect the historical legacy of Africa’s higher education systems, where social sciences have long been prioritised; and the relatively low representation of health and natural sciences underscores a need to rebalance support to include underrepresented disciplines. It is clear that English remains the dominant language of publication, supporting global reach, and only continuous, deliberate support for multilingual publishing would ensure linguistic equities.

Without coordinated investment in the ‘budding’ publishing research ecosystem, Africa-led investment in repositories, indexing services, and shared journal platforms, African research risks remaining peripheral in global knowledge systems. Learning from successful regional models such as Latin America’s SciELO and Redalyc, Africa needs to prioritise collective infrastructure development to ensure that its growing research output is visible, accessible, and impactful. At the same time, international actors could potentially support African leadership and African-led initiatives through resource mobilisation, partnership, and science diplomacy. A pan-African publishing strategy anchored in public institutions and regional networks will be key to strengthening Africa’s voice in global research.

## Ethical approval

This study did not involve human participants, animal subjects, or the use of identifiable personal data; therefore, ethical approval was not required. No institutional review board or ethics committee approval was sought, as the research relied solely on secondary data drawn from publicly available sources.

## Data Availability

Zenodo: List of African Publishers and associated journals. https://doi.org/10.5281/zenodo.16880223 ^43^. The project contains the following underlying data: Data-Africa research publishing landscape.xlsx Zenodo: Supplementary Materials Table 1–4: African Publishing Landscape. https://doi.org/10.5281/zenodo.17293320 ^44^. The project contains the following underlying data: Supplementary Material 1. Number of publishers, journals, and African countries covered by each source.docx Supplementary Material 2. Publishers and number of articles in their databases.docx Supplementary Material 3. Number of publishers and citation numbers mentioned in their databases.docx Supplementary Material 4. Table showing distribution of external publishers in African countries from Clarivate Analytics data.docx Data are available under the terms of the Creative Commons Attribution 4.0 International License (CC BY 4.0)
