# Supplementary material for: Africa’s research publishing landscape: examining journals, publishers, and the infrastructure behind them
Source: Open Res Afr. 2025 Oct 15;8:20. [Version 1] doi: 10.12688/openresafrica.16040.1 (PMC12775667; doi:10.12688/openresafrica.16040.1)
Supplement: Peer review history

# Reviewer report: Reviewer response for version 1

**DOI:** 10.21956/openresafrica.17215.r33054

**Published:** 2026-01-06

**Authors:** Abdelghani Maddi

**Affiliations:** 1 Sorbonne Université, CNRS, Paris, France

This article presents an ambitious and much-needed mapping of Africa’s scholarly journal publishing landscape, based on the integration of multiple data sources (DOAJ, AJOL, WoS, and others). The effort to consolidate heterogeneous datasets, manually verify publishers, and make the resulting data openly available represents a substantial contribution to the literature on scholarly communication and research infrastructure in the Global South.

 The manuscript is clearly written, methodologically transparent, and firmly anchored in relevant debates on open access, infrastructure dependency, and epistemic inequality. The descriptive results offer a very interesting insights into publisher typologies, geographic distribution, language use, and access models.

 However, while the empirical mapping itself is convincing, several key interpretations go beyond what the available data can robustly support. In particular, the notions of *fragmentation*, *regional concentration*, and *structural weakness* would benefit from a more cautious and analytically grounded treatment.

 Major comments

 1. Fragmentation is inferred without analysing publication volumes: A central claim of the paper is that African research publishing is “highly fragmented”, primarily based on the observation that most publishers operate only one or a few journals.

 While this descriptive finding is important, fragmentation cannot be meaningfully assessed using counts of publishers alone. In most regions of the world, including Europe and North America, scholarly publishing is characterised by:

- a very large number of formal publishers (societies, university presses, journal owners), and
- a strong concentration of *publication volumes* (articles, citations, revenues) within a small subset of actors.

In the present study, the authors explicitly note that article-level volume data are missing for the vast majority of publishers (nearly 90%). In the absence of such information, it is not possible to determine whether Africa’s publishing system is genuinely more fragmented than others, or whether it merely *appears* fragmented at the level of organisational counts.

 I therefore recommend:

- reframing fragmentation as a descriptive organisational pattern rather than a demonstrated structural condition; or
- explicitly acknowledging (in the limitations) that conclusions about fragmentation remain tentative in the absence of volume-based concentration measures.

Without this clarification, the argument risks conflating *numerical decentralisation* with *functional fragmentation*.

 2. Interpretation of regional concentration does not sufficiently account for linguistic and visibility biases

 The manuscript highlights the strong concentration of publishers and journals in Egypt, Nigeria, and South Africa, attributing this primarily to differences in research investment, institutional maturity, and population size. While these explanations are plausible and partly convincing, they are incomplete.

 Earlier sections of the paper rightly emphasise that:

- international databases systematically under-index African journals, and
- non-English publications are particularly disadvantaged in terms of visibility.

However, these well-documented linguistic and indexing biases are not explicitly mobilised when interpreting national and regional concentration patterns. This is problematic, given that:

- Nigeria and South Africa are predominantly anglophone systems, and
- Egyptian journals that achieve international visibility are overwhelmingly English-language outlets.

As a result, part of the observed concentration may reflect data visibility effects rather than underlying differences in publishing capacity or activity.

 I strongly encourage the authors to:

- explicitly discuss the potential role of linguistic bias in shaping national-level patterns; and
- distinguish more clearly between *observed concentration in indexed data* and *actual concentration in publishing activity*.

This would strengthen the analytical coherence of the manuscript and align the discussion with the methodological caveats raised elsewhere.

 3. The discussion of APC levels would benefit from a more context-sensitive interpretation. While the manuscript rightly notes that median APCs of around USD 250 are low when compared to fees charged by major commercial publishers in Europe and North America, this framing implicitly adopts a Global North benchmark.

 In many African countries, USD 250 may correspond to a full monthly salary or more for early-career researchers or lecturers, particularly in public universities. As a result, APCs that appear “modest” from a Northern perspective may still constitute a substantial economic barrier to participation in scholarly publishing for a large share of researchers.

 Consequently, low APCs should not be equated with affordability or inclusivity without additional evidence. Assessing inclusiveness would require data on:

- who is able to publish in these journals,
- the availability of institutional or external funding to cover APCs,
- the prevalence of fee waivers or informal exemptions.

I therefore recommend reframing the discussion to acknowledge that APC affordability is highly context-dependent, and that even relatively low APCs may reproduce inequalities within African research systems.

 Minor comments

- Terminology such as “research publishing”, “scholarly publishing”, and “journal publishing” is used somewhat interchangeably. Greater consistency would, in my opinion, improve the conceptual clarity.
- Some conclusions could be slightly softened to better reflect acknowledged data limitations, especially regarding representativeness.

Is the work clearly and accurately presented and does it cite the current literature?

Yes

If applicable, is the statistical analysis and its interpretation appropriate?

Yes

Are all the source data underlying the results available to ensure full reproducibility?

Yes

Is the study design appropriate and is the work technically sound?

Yes

Are the conclusions drawn adequately supported by the results?

Partly

Are sufficient details of methods and analysis provided to allow replication by others?

Yes

Reviewer Expertise:

Open Science, Research Integrity, Research Evaluation, Scientometrics

I confirm that I have read this submission and believe that I have an appropriate level of expertise to confirm that it is of an acceptable scientific standard, however I have significant reservations, as outlined above.

# Reviewer report: Reviewer response for version 1

**DOI:** 10.21956/openresafrica.17215.r33359

**Published:** 2025-12-31

**Authors:** Lai Ma

**Affiliations:** 1 University College Dublin, Dublin, Leinster, Ireland

The article makes a significant contribution by mapping Africa’s research publishing using numerous data sources. The findings about the number of small-scale publishing, linguistic diversity and the trends towards open access are very important for policies and initiatives pertaining to scholarly publishing in Africa and the rest of the world. The discussions about diamond open access and small-scale publishing in the humanities can benefit from the exchange of knowledge between regions. The need for visibility and infrastructure support would initiate constructive conversations for publishers with limited resources. 

 Major changes are not required, but a few very minor revisions may be useful. One is about the description of APCs in the Introduction: I assume that transformative (or “read-and-publish” or “transitional”) agreements have not been established between African institutions and major commercial publishers based in the Western hemisphere? This can be further elaborated with the lack of investment and funding in the research systems. A related question is whether the volume of research outputs in African or selected countries is proportional to R&D expenditure discussed in the second paragraph—though this may be beyond the scope of the article and can be examined in the future. 

 This sentence may be elaborated or clarified to avoid misinterpretation: “The remaining 3% of publishers were without clear country information or were based outside the continent” (Overview of publishers in Africa). I assume that the publishers without clear country information are based in the Africa continent, and those based outside the continent means that the editorial teams are based in Africa? 

 To the best of my knowledge, a similar comprehensive mapping exercise has not been done in Europe and North America, meaning that many journals in the arts and humanities and the social sciences are not known because of the limited coverage of AHSS in Web of Science and Scopus. Hence, the findings regarding disciplinary focus can say that the number of publishers in STEM are certainly small compared to the major “international” journals indexed in WoS and Scopus, but it would be difficult to make a comparison regarding AHSS journals due to the lack of data.

Is the work clearly and accurately presented and does it cite the current literature?

Yes

If applicable, is the statistical analysis and its interpretation appropriate?

I cannot comment. A qualified statistician is required.

Are all the source data underlying the results available to ensure full reproducibility?

Yes

Is the study design appropriate and is the work technically sound?

Yes

Are the conclusions drawn adequately supported by the results?

Yes

Are sufficient details of methods and analysis provided to allow replication by others?

Yes

Reviewer Expertise:

scholarly communication; open research

I confirm that I have read this submission and believe that I have an appropriate level of expertise to confirm that it is of an acceptable scientific standard.

# Reviewer report: Reviewer response for version 1

**DOI:** 10.21956/openresafrica.17215.r32715

**Published:** 2025-11-04

**Authors:** Mikael Laakso

**Affiliations:** 1 Tampere University, Tampere, Tampere, Finland

The study identifies and approaches a substantial and persistent research gap in the data and knowledge we have about scholarly journal publishing in Africa. As many previous studies have shown over the last decade, the most commonly used international bibliometric data sources fail to provide sufficient coverage for scholarly journals published across the African continent.  Concerning how this existing research is summarised and treated in the introduction the study the article manuscript is executed well, the gap in the research is clearly identified and motivated using relevant existing literature.

 The study formulates four ambitious research questions that it seeks answers to:

- RQ1: What is the current size and geographic distribution of the research publishing sector in African countries?
- RQ2: What kinds of research disciplines do African research platforms publish?
- RQ3: What languages do African research publishers publish in?
- RQ4: How open or closed is African research publishing?

Concerning these I have two comments that I would like the authors to consider in a potential revision:

 1. Since the study is focusing its data collection and analysis to only scholarly journals I would suggest that the questions would be adjusted to account for this, making them a bit narrower and more precise to the exact scope of the empirical part of the study.

  2. While the study incorporates some local datasets in addition to utilizing what is available from international sources, it should not be considered as a definitive population which the study now makes it seem throughout the manuscript. There are certainly many more African journals out there that for one reason or another is not documented within these datasets. I would like this to be taken better into account in the text, and not just as a couple of sentences within the limitations since I think the issue of discoverability is still not completely solved, even though this is the most comprehensive study to date. So essentially I am calling out for a better separation between what can be said concerning 1) development and changes in the publishing landscape itself, and 2) changes and limitations that relate to lacking discovery and indexation of African scholarly journals.

 The methods section is clear and the authors have put in a lot of meticulous effort in manually classifying the publishers and visiting journal websites to collect additional or missing metadata that could not be obtained any other way. Transparency in documentation is strong. I applaud the authors for making the research dataset openly available, these kinds of actions will help build better discoverability and even broader and more comprehensive studies in the future, getting rid of the need for every project to start from scratch aggregating the metadata for the same publication outlets.

 The results of the descriptive mapping study are provided through nicely designed visualizations and tables, providing information on the dimensions one would expect to see based on the perspectives defined through the research questions (but please keep in mind the comments concerning them I mentioned earlier). Overall I have nothing major to comment with regards to results presentation and discussion beyond what I already have said, the study provides a valuable puzzle piece for research and policy to have a better understanding of the publishing activity on the African continent. My suggestions for minor revisions do to necessitate any changes to the empirical part of the study, I am mainly calling for a more tightly defined research questions and a more humble formulation on how much of all scholarly journal publishing activity this study is able to capture of everything that is happening on the continent.

Is the work clearly and accurately presented and does it cite the current literature?

Yes

If applicable, is the statistical analysis and its interpretation appropriate?

Yes

Are all the source data underlying the results available to ensure full reproducibility?

Yes

Is the study design appropriate and is the work technically sound?

Yes

Are the conclusions drawn adequately supported by the results?

Partly

Are sufficient details of methods and analysis provided to allow replication by others?

Yes

Reviewer Expertise:

scholarly publishing, scholarly communication, open access, open sciece

I confirm that I have read this submission and believe that I have an appropriate level of expertise to confirm that it is of an acceptable scientific standard, however I have significant reservations, as outlined above.
